# Supplementary material for: Actin-Resistant DNase1L2 as a Potential Therapeutics for CF Lung Disease
Source: Biomolecules. 2021 Mar 10;11(3):410. doi: 10.3390/biom11030410 (PMC8002113; doi:10.3390/biom11030410)
Supplement: Supplementary file 1 [file biomolecules-11-00410-s001.zip › DNase1L2_suplementary_figures.pdf]

Name	Transcript ID	bp	Protein	Translation ID	Biotype	CCDS	UniProt Match	RefSeq Match	Flags
DNASE1L2-201	ENST00000320700.10	1349	299aa	ENSP00000316938.	Protein coding	CCDS42105	Q92874-1	NM_001374.3	TSL:1, GENCODE basic, APPRIS P4, MANE Select v0.91
DNASE1L2-202	ENST00000382437.8	1222	278aa	ENSP00000371874.	Protein coding	-	Q92874-2	-	TSL:1, GENCODE basic, APPRIS ALT2
DNASE1L2-203	ENST00000564065.5	2194	299aa	ENSP00000454562.	Protein coding	CCDS42105	Q92874-1	-	TSL:1, GENCODE basic, APPRIS P4
DNASE1L2-204	ENST00000567494.5	1301	299aa	ENSP00000455358.	Protein coding	CCDS42105	Q92874-1	-	TSL:1, GENCODE basic, APPRIS P4
DNASE1L2-205	ENST00000569184.1	762	254aa	ENSP00000455478.	Protein coding	-	H3BPU8	-	CDS 5' and 3' incomplete, TSL:3
DNASE1L2-206	ENST00000613572.4	1260	278aa	ENSP00000482627.	Protein coding	-	Q92874-2	-	TSL:5, GENCODE basic, APPRIS ALT2

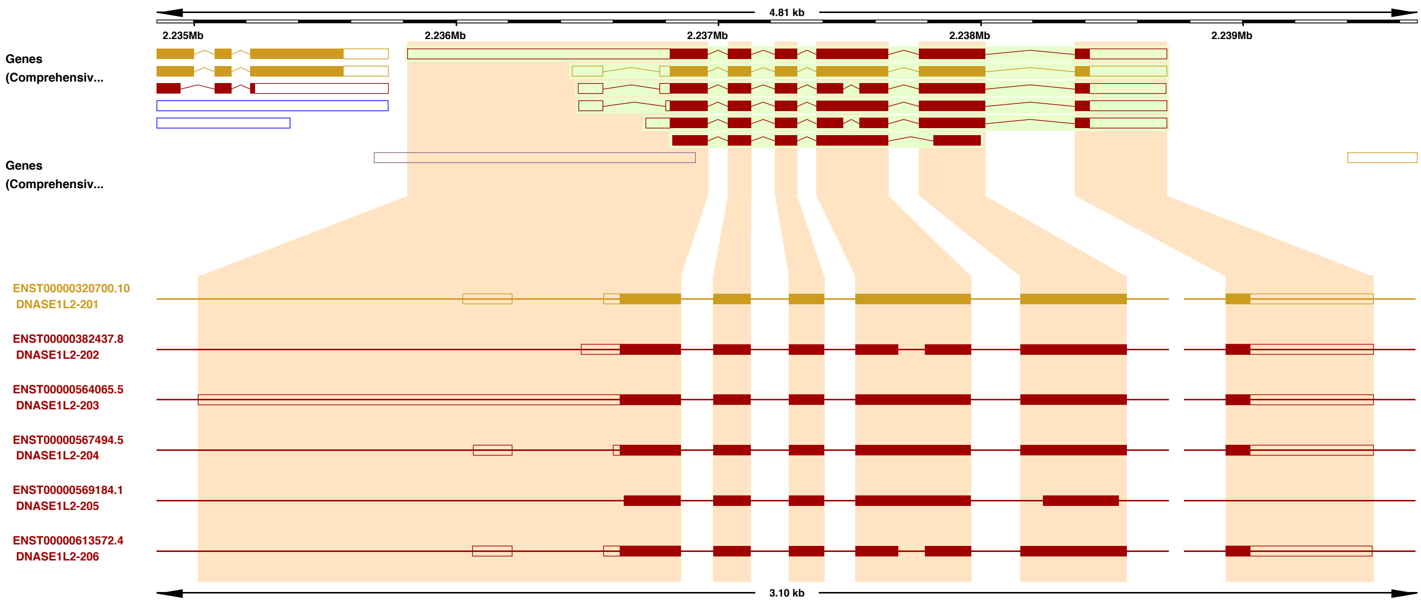

Figure S1. **Transcripts of human DNase1L2 gene annotated by the ENSEMBL database.** Predicted transcripts of the human DNase1L2 gene (ENSG00000167968) in ENSEMBL Rel. 101. Boxes represent exon regions with the coding sequence highlighted in colors. Transcripts corresponding to the L (yellow), S (orange), and incomplete (gray) isoforms are highlighted in the transcript table.

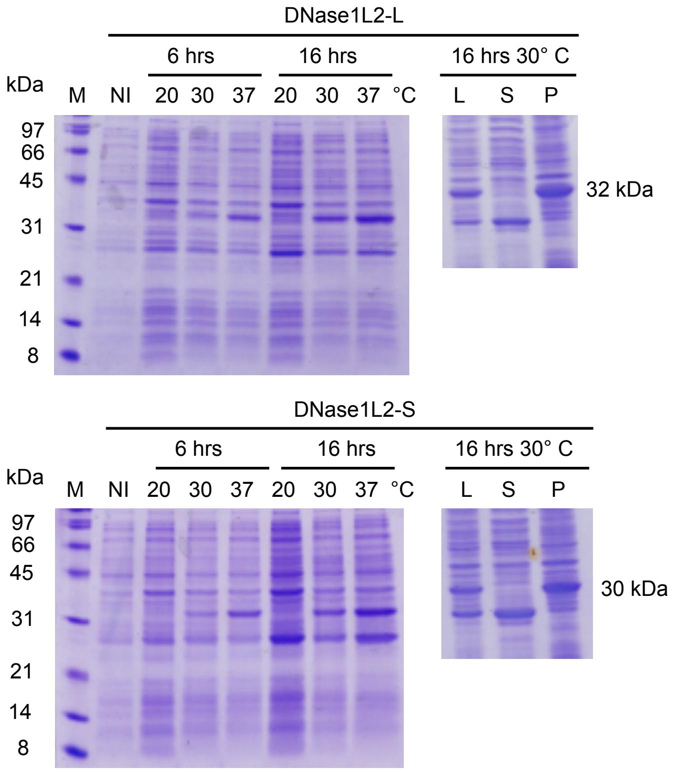

Figure S2. **Recombinant expression of DNase1L2-L and DNase1L2-S in *E. coli*.** SDS-PAGE analysis of expression (left panels) and solubility (right panels). Expression was assayed at different temperatures (20, 30 and 37°C) and times (6 and 16 hours) after induction with IPTG. The evaluation of solubility was performed for every culture yielding positive induction and the results were similar: the recombinant proteins were found in the insoluble fraction. M: Marker, NI: Not Induced total cell extract, L: total cell extract after Lysis, S: soluble fraction (Supernatant), P: insoluble fractions (Pellet).

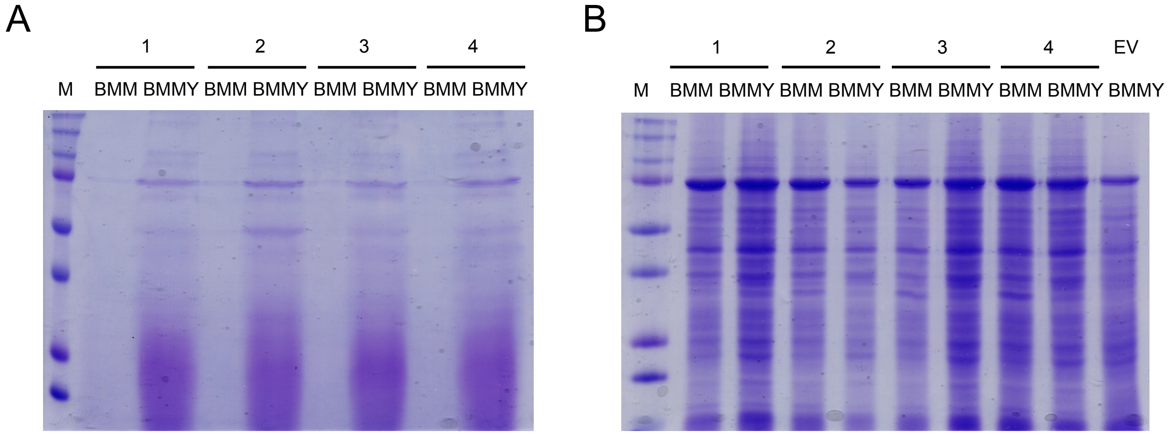

Figure S3. **Recombinant expression of DNase1L2-L in *P. pastoris*.** SDS-PAGE analysis of (A) extracellular and (B) intracellular proteins produced by *P. pastoris* GS115 transformed with pPIC9K-DNase1L2-L (four clones, 1-4, are shown) or with an empty vector (EV) in different media (BMM and BMMY). M: Marker.

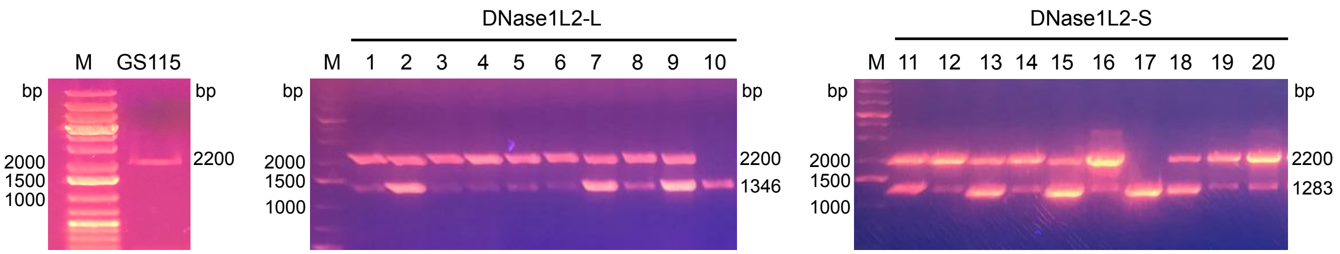

Figure S4. **Integration of DNase1L2-L and DNase1L2-S genes into the *P. pastoris* GS115 genome confirmed by PCR using 5'AOX1 and 3'AOX1 primers.** M: DNA ladder, GS115: PCR product of untransformed *P. pastoris* showing one band of 2200 bp that corresponds to the size of the AOX1 in the *P. pastoris* genome, lanes 1-10: PCR products of *P. pastoris* transformed with pPIC9K-DNase1L2-L [lanes 1-9 show two bands of 2200 bp and 1346 bp that correspond respectively to the AOX1 and the DNase1L2-L integrated at the *his4* locus (Mut+); lane 10 shows one band of 1346 bp that corresponds to the DNase1L2-L integrated at the AOX1 locus (MutS)], lanes 11-19: PCR products of P. *pastoris* transformed with pPIC9K-DNase1L2-S [lanes 11-16 and lanes 18-20 show two bands of 2200 bp and 1283 bp that correspond respectively to the AOX1 and the DNase1L2-S integrated at the *his4* locus (Mut+); lane 17 shows one band of 1283 bp that corresponds to the DNase1L2-S integrated at the AOX1 locus (MutS)].

Name: Hsapiens_DNase1L2 Length: 278

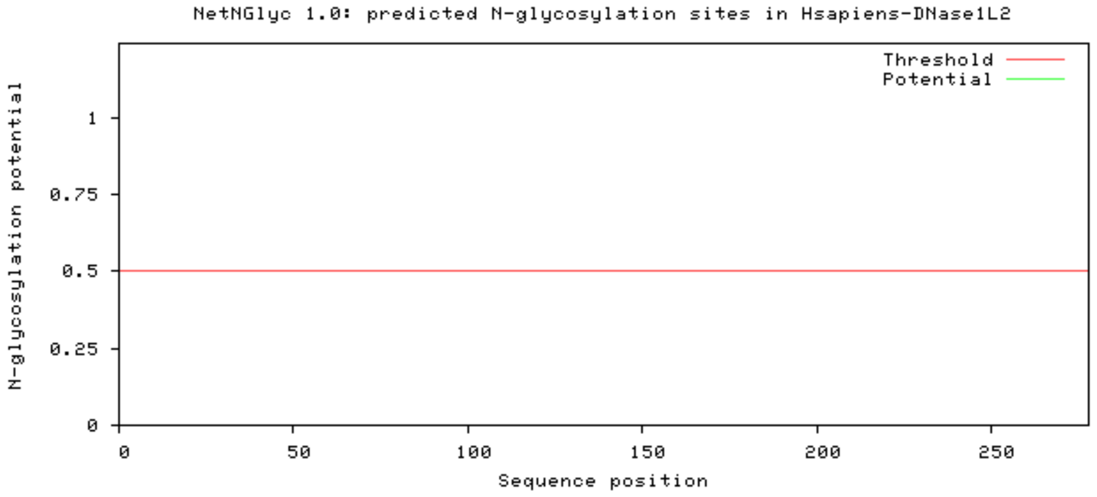

No sites predicted in this sequence.

Name: Hsapiens_DNase1 Length: 282

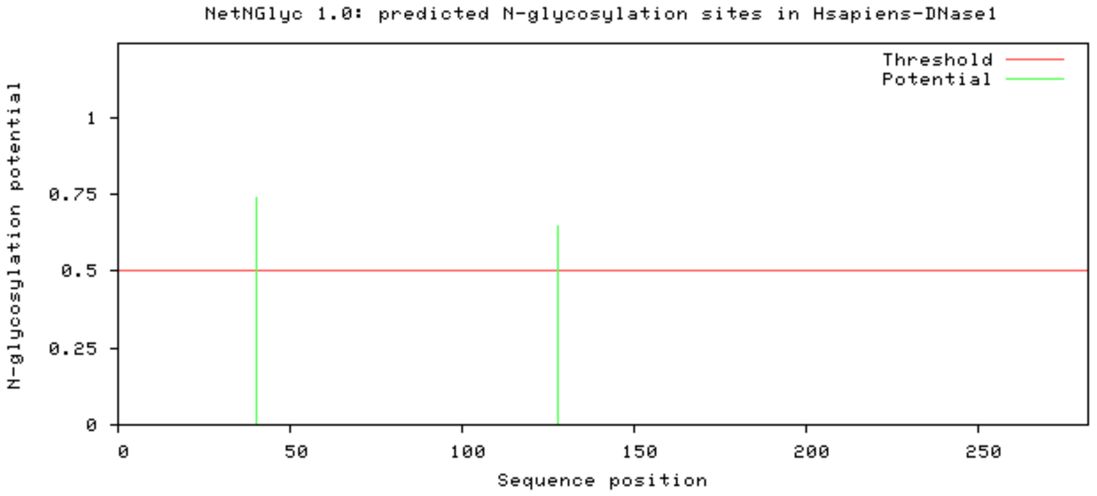

SeqName	Position		Potential	Jury agreement	N-Glyc result
Hsapiens_DNase1	40	NATL	0.7360	(9/9)	++
Hsapiens_DNase1	128	NDTF	0.6456	(8/9)	+

Figure S5. **Prediction of N-glycosylation pattern in human DNase1L2 and DNase1 by NetNGlyc 1.0.** The output graphs show N-glycosylation potential versus amino acids position in the sequence. The two N-glycosylation sites of DNase1 are not conserved in DNase1L2 (see Figure 4B).

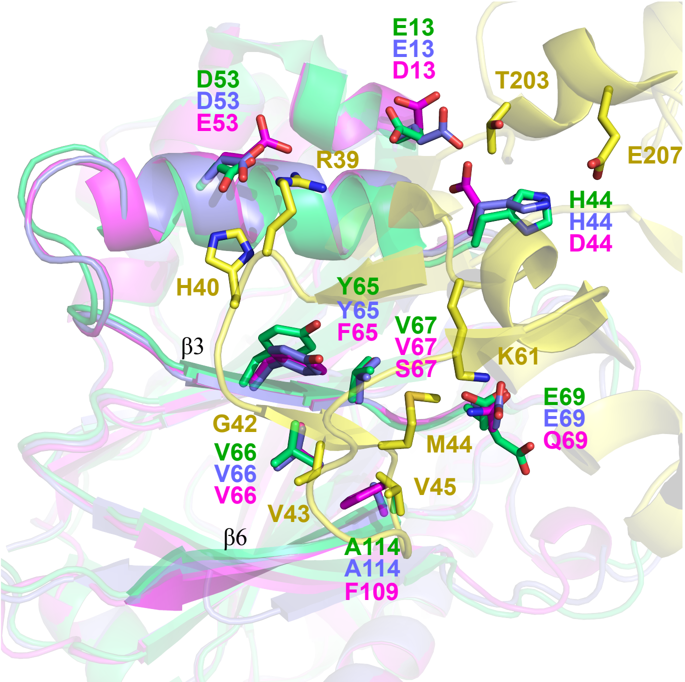

Figure S6. **DNase1/DNase1L2-actin interface.** Superimposition of DNase1L2 model (pink carbons) with the crystal structures of human DNase1 (green carbons) and bovine DNase1 (violet carbons) in complex with actin(yellow carbons). The key residues involved in interactions are shown in sticks and labeled. β-strands of DNase1L2 and DNase1 interacting with actin are labeled.

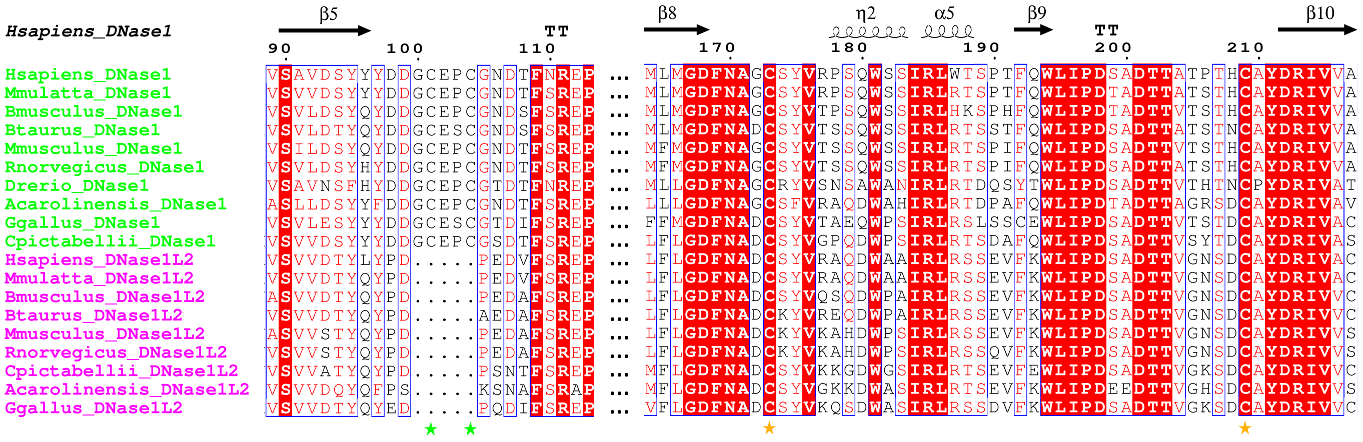

Figure S7. **Conservation of only one cysteine pair in DNase1L2.** Portion of a multiple alignment of vertebrate DNase1 and DNase1L2 sequences showing a cysteine pair conserved in all DNase1 and DNase1L2 proteins (yellow stars) and a cysteine pair conserved only in DNase1 (green stars), because of a deletion of five codons in DNase1L2.
