# Supplementary material for: Actin-Resistant DNase1L2 as a Potential Therapeutics for CF Lung Disease
Source: Biomolecules. 2021 Mar 10;11(3):410. doi: 10.3390/biom11030410 (PMC8002113; doi:10.3390/biom11030410)
Supplement: Supplementary file 1 [file biomolecules-11-00410-s001.zip › DNase1L2_suplementary_tables.pdf]

Table S1. **Ensembl accession number, classification and name of DNase1L2 proteins included in the multiple alignment.**

**Organism**	**Classification**	**Name[a]**	**Accession n°**
H. sapiens	Mammalia	DNase1L2-L	ENSP00000316938.5
H. sapiens	Mammalia	DNase1L2-S	ENSP00000371874.4
P. troglodytes	Mammalia	DNase1L2-L	ENSPTRP00000013061
G. gorilla	Mammalia	DNase1L2-S	ENSGGOP00000013907
M. mulatta	Mammalia	DNase1L2-S	ENSMMUP00000053522
A. nancymaae	Mammalia	DNase1L2-L	ENSANAP00000013147
O. cuniculus	Mammalia	DNase1L2-S	ENSOCUP00000016098
T. truncatus	Mammalia	DNase1L2-L	ENSTTRP00000007777
B. taurus	Mammalia	DNase1L2-S	ENSBTAP00000013142
P. vampyrus	Mammalia	DNase1L2-L	ENSPVAP00000004824
E. telfairi	Mammalia	DNase1L2-L	ENSETEP00000007838
R. norvegicus	Mammalia	DNase1L2-S	ENSRNOP00000012203
M. musculus	Mammalia	DNase1L2-S	ENSMUSP00000085862
L. africana	Mammalia	DNase1L2-S	ENSLAFP00000027192
M. monoceros	Mammalia	DNase1L2-S	ENSMMNP00015009148
M. domestica	Mammalia	DNase1L2-S	ENSMODP00000019845
G. gallus	Sauropsida	DNase1L2-S	ENSGALP00000064712
A. carolinensis	Sauropsida	DNase1L2-S	ENSACAP00000000576
C. pictabellii	Sauropsida	DNase1L2-S	ENSCPBP00000015855
P. major	Sauropsida	DNase1L2-S	ENSPMJP00000013429

[a]Name in accordance with the presence (DNase1L2-L) or absence (DNase1L2-S) of the P-rich region (see Figure 1A)

Table S2. **Viscosity measurements of CF artificial mucus after incubation with DNase1L2 or rhDNase in the presence or absence of actin (0.2 mg/ml).**

	Viscosity mPa·s (mean ± SD)		
	90 s	300 s	600 s
no enzyme	9783 ± 1656	9667 ± 1457	ND
no enzyme + Actin	8141 ± 689	8171 ± 932	ND
DNase1L2	1847 ± 263	603 ± 155	236 ± 40
DNase1L2 + Actin	4649 ± 960	2570 ± 865	1292 ± 446
rhDNase	410 ± 58	18 ± 16	ND
rhDNase + Actin	9330 ± 1537	8469 ± 2068	6666 ± 2747
rhDNase* + Actin	8606 ± 1377	4925 ± 2663	1432 ± 1410

Enzyme concentration was: DNase1L2, 20 ng/μl - 5 U; rhDNase, 2 ng/μl - 5 U; rhDNase*, 20 ng/μl - 50 U
